# Different glucose analyzers report different glucose concentration values in term newborns

**DOI:** 10.3389/fped.2022.933508

**Published:** 2022-09-21

**Authors:** Rina P. Duke, Shasha Bai, Joshua A. Bornhorst, Nahed O. ElHassan, Jeffrey R. Kaiser

**Affiliations:** ^1^Department of Pediatrics (Neonatology), Lehigh Valley Reilly Children's Hospital, Allentown, PA, United States; ^2^Pediatrics Biostatistics Core, Department of Pediatrics, Emory University School of Medicine, Atlanta, GA, United States; ^3^Department of Laboratory Medicine and Pathology, Mayo Clinic, Rochester, MN, United States; ^4^Department of Pediatrics (Neonatal-Perinatal Medicine), University of Arkansas for Medical Sciences, Little Rock, AR, United States; ^5^Departments of Pediatrics (Neonatal-Perinatal Medicine) and Obstetrics and Gynecology, Penn State Children's Hospital, Hershey, PA, United States

**Keywords:** neonatal hypoglycemia, newborn, glucose, glucose analyzer, guideline

## Abstract

**Background:**

The American Academy of Pediatrics and Pediatric Endocrine Society neonatal hypoglycemia guidelines based their glucose concentration treatment thresholds on studies that predominantly used Beckman and Yellow Springs Glucose Oxidase Analyzers. Currently, a majority (76%) of U.S. hospital laboratories utilizing glucose oxidase methodology use Vitros^®^ Glucose Analyzers. However, a bias of ~+5% between glucose concentrations from Beckman vs. Vitros Glucose Analyzers has been reported; this could have a clinically significant effect when using published guideline treatment thresholds.

**Methods:**

To determine if there is similar instrument bias between Beckman and Vitros Analyzers in reported glucose concentrations from term newborns, we compared plasma glucose concentrations measured within the first 3 h after birth by Beckman vs. Vitros Analyzers in a total of 1,987 newborns (Beckman *n* = 904, Vitros *n* = 1,083). Data were fit using nonlinear cubic spline models between collection time and glucose concentration.

**Results:**

The non-linear patterns of initial glucose concentrations (during the first 3 h after birth) as measured by Beckman and Vitros Analyzers paralleled each other with no overlap of the fit spline curve 95% confidence intervals, with an approximate +5 mg/dL constant bias. Additionally, in method comparison studies performed in the Chemistry Laboratory on adult samples, there was a +4.2-7.4 mg/dL measured glucose bias for the Beckman vs. Vitros Analyzer.

**Conclusion:**

Glucose concentrations from term, appropriate size for gestational age newborns were about 5 mg/dL higher when measured by Beckman vs. Vitros Analyzers. Perhaps, concentrations of 45 mg/dL reported from Beckman Analyzers may be equivalent to 40 mg/dL from Vitros Analyzers. When managing neonatal hypoglycemia, it is important to know which analyzer was used and whether adjusting for potential instrument bias is necessary when following published guidelines.

## Introduction

Transient hypoglycemia is the most common metabolic disturbance in newborns, affecting up to 15% of term infants (1, 2). While severe, recurrent, and prolonged neonatal hypoglycemia has been linked to brain injury and poor neurodevelopmental outcomes (3–6), many knowledge gaps persist regarding *transient* neonatal hypoglycemia. Some controversies regarding our current understanding of transient hypoglycemia include: 1) whether it is a normal physiological phenomenon or is associated with cognitive impairment and brain injury, 2) the glucose concentration that defines it, 3) which glucose concentration thresholds to use for treatment, 4) specific treatment strategies, and 5) whether treatment even improves outcomes (2, 7). Addressing these knowledge gaps requires confidence in reported glucose concentration measurements when using published guidelines to determine which newborns to screen, treat, and ultimately separate from their mothers when treatment requires transfer from the Newborn Nursery to the Neonatal Intensive Care Unit (NICU).

The American Academy of Pediatrics (AAP) (8) and the Pediatric Endocrine Society (PES) (9) published different glucose concentration thresholds for managing neonatal hypoglycemia. The AAP recommends treating newborns with glucose concentrations < 25 mg/dL during the first 4 h and < 35 mg/dL between 4 to 24 h, and maintaining glucose concentrations >45 mg/dL between 24 to 48 h after birth (8, 10). In contrast, the PES recommends a glucose concentration target of >50 mg/dL during the first 48 h and >60 mg/dL thereafter (9).

Recommendations by the AAP (8) and the PES (9) are mostly based on studies from the 1970s−2000s that used glucose analyzers employing the glucose oxidase method [Beckman Glucose Analyzer [Beckman Coulter, Inc., Brea, CA] (11–15) and Yellow Springs Instrument Glucose Analyzer [Yellow Springs, OH] (16, 17)]. Although the Yellow Springs Instrument has now been discontinued, glucose concentration values from the Yellow Springs Analyzers correlated well (r = 0.997) with those obtained from Beckman Analyzers (18). This is particularly relevant because the majority (76%) of laboratories in the U.S. that *now* utilize glucose oxidase analyzers use Vitros^®^ Analyzers (Ortho Clinical Diagnostics, Raritan, NJ) (19).

The use of the Vitros Analyzer slide technology for glucose measurement has potential advantages (smaller analytical sampling volumes and less susceptibility to interference from lipidemia) (20) for glucose determination vs. other methods, such as the Beckman Analyzer. As a result, the Vitros Analyzer may consistently exhibit small yet clinically important glucose concentration differences (or systematic bias) relative to other analyzers. For example, a study evaluating standardization among different chemistry laboratory analyzers (21) observed (using a glucose concentration standard of 100 mg/dL), a relative bias of +2% and −3% respectively for Beckman and Vitros Analyzers relative to the same standardized material, which equates to an estimated positive bias of 5 mg/dL of Beckman Analyzer glucose measurements relative to the Vitros Analyzer. Thus, we hypothesize that in comparable newborn patient samples, Vitros Glucose Analyzers will report lower plasma glucose concentrations than Beckman Analyzers (21).

In this observational study, we compared the initial plasma glucose concentrations within the first 3 h after birth in term newborns as measured by Beckman vs. Vitros Analyzers.

## Methods

### Participants

The study included term (22) (≥37– < 42 weeks' gestation) newborns born in 1998 and 2008 at the University of Arkansas for Medical Sciences who were appropriate size for gestational age (birth weight between the 10^th^ and 90^th^ percentile) (23), non-asphyxiated (5-min Apgar score ≥7), and not infants of diabetic mothers (pre-existing or pregnancy-related) if they had ≥1 plasma glucose specimen obtained within the first 3 h after birth. The study excluded newborns with major congenital anomalies, inborn errors of metabolism, or chromosomal abnormalities. The University of Arkansas for Medical Sciences Institutional Review Board approved the study and waived informed consent.

### Data collection

Glucose specimens were collected within 3 h of birth and analyzed in the Pediatric Laboratory within the NICU and near the Newborn Nursery. We obtained dates and times for specimen collection of lithium-heparin plasma glucose concentrations (mg/dL). Obstetrical gestational age estimates were obtained from the mothers' medical records and estimated based on, in declining order: last menstrual period > uterine size at the first obstetrical examination > early trimester ultrasound dating > uterine size at follow-up examinations. We abstracted the neonatal gestational age estimate (24) from newborns' medical records. If the neonatal estimate differed by >2 weeks from the obstetrical estimate, we used the neonatal estimate. We used a uniform characterization of gestational age (e.g., 38 weeks of gestation applied to newborns identified as “38+ weeks' gestation” or “38 weeks, 0 to 6 days of gestation”).

### Laboratory glucose concentration measurement during the study period

During the 10-year course of this study, the University of Arkansas for Medical Sciences used different generations of two types of laboratory analyzers for measuring plasma glucose concentrations in the NICU and Newborn Nursery. In 1998, glucose measurements were performed using the Beckman Glucose Analyzer 2, which used the glucose oxidase method in concert with the Beckman Coulter Oxygen electrode (25). This glucose analyzer electrode is functionally equivalent to those employed in later Beckman Coulter Synchron instruments, including the LX-20 and DxC 800 models. In August 2001, the Vitros 250 Chemistry Analyzer was put into service for measurement of plasma glucose concentrations. This instrument employs a glucose slide technology using the glucose oxidase reaction (20). In 2007, this instrument was upgraded to the functionally identical Vitros 350 instrument, which was used in 2008.

While the hexokinase method of blood glucose measurement is regarded as more specific than the glucose oxidase method, almost all current glucose analysis is performed by one of these two enzymatic methods (26, 27). Despite calls for a “gold standard” test to be adopted for glucose measurements, the National Academy of Clinical Biochemistry guidelines did not recommend the use of one of these methods over the other, in part due to a lack of consistent bias perceived between methods (27–29). While a “gold standard” analysis for accurate testing for glucose by isotope dilution or mass spectrometry have been proposed, in practice, the use of this mass spectrometry method is not widespread and may not be able to provide adequate testing turn-around times (29, 30).

### Chemistry laboratory comparison of glucose concentrations between Beckman and Vitros analyzers

We performed two representative glucose concentration method comparative studies in the main hospital Chemistry Laboratory using glucose specimens on discarded patient samples. We simultaneously measured fresh lithium-heparin plasma samples in BD Microtainer^®^ tubes (Becton, Dickinson and Company, Franklin Lakes, NJ) in each analyzer. The first glucose comparison study (*n* = 29) in 2006 showed a constant positive bias of 7.4 mg/dL (31) for the Beckman Coulter Synchron LX-20 instrument vs. the Vitros 250 Chemistry Analyzer. The second glucose comparison experiment (*n* = 20) in 2015 showed a similar positive constant bias of 4.2 mg/dL for the Beckman Coulter Synchron DxC 800 instrument vs. the Vitros 350 Chemistry Analyzer.

### Statistical analysis

Summary statistics for clinical and demographic information from newborns and mothers were determined. We summarized categorical data as frequencies (% of the total) and interval/ordinal data as medians (interquartile range). For differences between newborns born during the Beckman and Vitros eras, we used the chi-square test for categorical variables, and the Mann Whitney U test for interval variables.

To analyze the agreement between glucose concentrations (simultaneously obtained from the same patient sample) reported from two glucose analyzers by the University of Arkansas for Medical Sciences Chemistry Laboratory, we determined the mean differences (i.e., the bias) using the Bland-Altman method (31).

Since the relationship between initial glucose concentration and collection time was expected to be non-linear (32), we used restricted cubic spline models, a special type of regression modeling technique, to relax the strict linear assumption and to capture the smooth non-linear trend we expected to see in the data. While cubic spline analysis is not one of the most standard statistical techniques encountered in pediatric research, it is very familiar to pediatricians and neonatologists because it was used to generate newborn growth curves (33, 34). This model allows prediction of average glucose levels to either increase or decrease as the collection time increases. We set the number of knots to 5, a sufficient number used to model most data. We performed all statistical analyses with Stata 15.0 (College Station).

## Results

### Descriptive characteristics

Descriptive and comparative statistics for newborns who had plasma glucose concentrations measured by Beckman (*n* = 904) vs. Vitros Analyzers (*n* = 1,083) are shown in Table 1.

**Table 1:** Demographic and birth characteristics of term newborns who had plasma glucose concentrations measured by Beckman vs. Vitros analyzers.

**Characteristics**	**Beckman**	**Vitros**	***p***
	*N* = 904	*N* = 1,083	
Year born	1998	2008	
Gestational age, median (IQR), weeks	39.0 (38.0, 40.0)	39.0 (38.0, 40.0)	0.003†
Birth weight, median (IQR), g	3,282 (3,068, 3,545)	3,335 (3,066, 3,583)	0.276†
Race, *n* (%)			< 0.001*
Black	416 (46.0)	283 (26.1)	
White	422 (46.7)	436 (40.4)	
Hispanic	47 (5.2)	312 (28.8)	
Asian	19 (2.2)	52 (4.8)	
Female sex, *n* (%)	429 (47.5)	510 (47.1)	0.871*
Apgar 5 mins, *n* (%)			0.039*
7 8 9 10	40 (4.4) 147 (16.3) 687 (76.0) 27 (3.0)	39 (3.6) 136 (12.6) 883 (81.5) 25 (2.3)	
Time to specimen collection, median (IQR), minutes	93 (75, 115)	101 (79, 126)	< 0.001†
Glucose concentration, median (IQR), mg/dL	59 (50, 68)	53 (46, 62)	< 0.001†

*Chi-Square test. †Mann Whitney U. IQR, interquartile range.

While birth weight was not different between the two study groups, gestational age was significantly different, although the difference was not clinically meaningful. There were fewer Black newborns and more Hispanic newborns in the Vitros era. Lastly, while the time from birth to specimen collection and the Apgar scores at 5 mins were statistically different, the differences were also not clinically meaningful.

### Comparison of glucose concentrations

**Figure 1:**
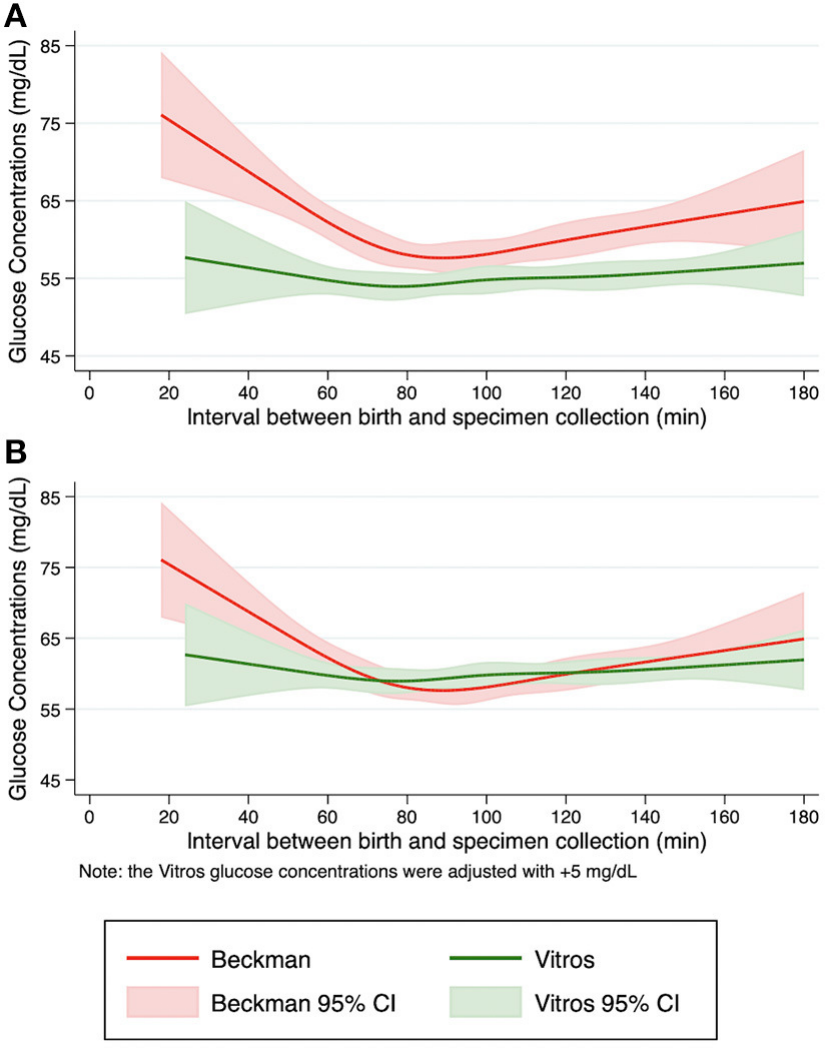
Pattern of fitted glucose concentrations during the first 3 h after birth. Restricted cubic spline curves were fit from initial glucose concentrations from term, appropriate size for gestational age newborns in the Beckman and Vitros Analyzers. **(A)** Fitted glucose concentrations and 95% confidence intervals from newborns using Beckman and Vitros Analyzers. **(B)** Fitted glucose concentrations and 95% confidence intervals from newborns using Beckman and Vitros Analyzers, after 5 mg/dL was added to the Vitros glucose concentration spline curve.

To compare the glucose concentration patterns between Beckman and Vitros Analyzers, the fit glucose concentrations and the 95% confidence intervals by specimen collection time are shown in Figure 1A. The non-linear pattern of initial glucose concentrations generally paralleled each other (constant bias) with no overlap of the spline curve or the 95% confidence intervals, with glucose concentrations ~5 mg/dL lower when measured by Vitros vs. Beckman Analyzers. Additionally, when a conservative value of 5 mg/dL was added to the Vitros Analyzer glucose pattern to mitigate this observed bias, the Beckman and Vitros Analyzer glucose concentration patterns overlapped (Figure 1B).

## Discussion

There are many continued controversies, knowledge gaps, disagreements, and lack of consensus among researchers and clinicians about neonatal hypoglycemia that persist despite over 4,000 articles published in the world literature. However, before we can fill any of these knowledge gaps and reach consensus, we must be confident in the commutability of reported glucose concentration measurements, and what observed bias may mean when following published guidelines. We observed clear differences in patterns of initial plasma glucose concentrations during the first 3 h after birth in term, appropriate size for gestational age, non-asphyxiated, and non-infants of diabetic mother newborns when measured by Beckman and Vitros Analyzers. The small (~5 mg/dL) yet noteworthy and important difference in plasma glucose concentrations observed in this study is consistent with both the known absolute glucose concentration bias difference of about 5 mg/dL (at 100 mg/dL) between Beckman and Vitros Analyzers (21), and with our own internal comparisons (4.2–7.4 mg/dL bias) between the glucose analyzers. Moreover, when a conservative constant value of 5 mg/dL was added to the Vitros glucose concentration spline curve, the curves and 95% confidence intervals from both analyzers overlapped. Our observations add yet additional complexity and controversy to this topic, specifically about which glucose concentration thresholds and targets should be recommended when screening for and treating neonatal hypoglycemia.

Several strengths of our study warrant mention. First, and while some consider it controversial in itself, the University of Arkansas for Medical Sciences has a universal newborn glucose screening policy that has provided us with comparative glucose concentrations from all, not at-risk, term newborns to perform this study. Additionally, each comparative group included about a thousand newborns. The glucose oxidase method was used in both laboratory analyzers, rather than less precise point-of-care glucometers. Importantly, because the laboratory was within the NICU and close to the Newborn Nursery, the time from specimen collection to result was exceedingly short, preventing glucose utilization from the red blood cells, so that reported concentrations were relatively reflective of the newborns when they were collected. Additionally, glucose specimens were obtained at similar times between the two study groups. Our study, however, has some limitations. This was an observational study conducted in a single center. Some of the baseline characteristics of the study groups were statistically different; however, the differences were not clinically relevant. There was also a statistically significant difference in race, with the Vitros group comprising fewer Black and more Hispanic newborns compared to the Beckman group. It is possible that this difference in race contributed to the lower glucose concentrations in the Vitros group; however, there are no studies linking racial differences to differences in glucose concentrations.

## Conclusion

Our observation that the initial glucose concentrations measured using Beckman Analyzers are about 5 mg/dL higher compared to those using Vitros Analyzers is an important empirical example of the systematic bias in these instruments. Therefore, previous guidelines about glucose thresholds and targets recommended from the AAP and PES need to be re-evaluated because they were primarily based on expert opinion using evidence from Beckman glucose analyzers (11, 13–15, 18, 35–37), which in the U.S. have been slowly replaced by the use of Vitros Analyzers nowadays. For example, a glucose concentration of 45 mg/dL reported from a Beckman Analyzer may be equivalent to 40 mg/dL reported from a Vitros Analyzer. This would significantly change the evaluation and treatment of neonatal hypoglycemia and have a huge impact on the care of newborns throughout the world. With an updated guideline, fewer newborns would be considered “hypoglycemic” and receive multiple needle sticks, and be separated from their mothers when transferred from the Newborn Nursery to NICU for treatment of neonatal hypoglycemia. Perhaps, when using the AAP or PES hypoglycemia management guidelines, glucose concentration treatment thresholds should be decreased by 5 mg/dL at institutions using Vitros Analyzers until more comprehensive glucose standardization is achieved. At the very least, clinicians need to be aware and mindful of which glucose analyzer is being used in their institutions and whether adjusting for potential instrument bias is necessary.

## Data availability statement

The raw data supporting the conclusions of this article will be made available by the authors, without undue reservation.

## Ethics statement

The studies involving human participants were reviewed and approved by University of Arkansas for Medical Sciences Institutional Review Board. Written informed consent from the participants' legal guardian/next of kin was not required to participate in this study in accordance with the national legislation and the institutional requirements.

## Author contributions

JK, SB, JB, and NE contributed to conception and design of the study and wrote sections of the manuscript. RD wrote the first draft of the manuscript. All authors contributed to manuscript revision, read, and approved the submitted version.

## Conflict of interest

The authors declare that the research was conducted in the absence of any commercial or financial relationships that could be construed as a potential conflict of interest.

## Publisher's note

All claims expressed in this article are solely those of the authors and do not necessarily represent those of their affiliated organizations, or those of the publisher, the editors and the reviewers. Any product that may be evaluated in this article, or claim that may be made by its manufacturer, is not guaranteed or endorsed by the publisher.

## References

[1] Kaiser JR Bai S Gibson N Holland G Lin TM Swearingen CJ. Association between transient newborn hypoglycemia and fourth-grade achievement test proficiency: a population-based study. JAMA Pediatr. (2015) 169:913–21. 10.1001/jamapediatrics.2015.1631 26301959

[2] Hay WW Jr Raju TN Higgins RD Kalhan SC Devaskar SU. Knowledge gaps and research needs for understanding and treating neonatal hypoglycemia: workshop report from Eunice Kennedy Shriver National Institute of Child Health and Human Development. J Pediatr. (2009) 155:612–7. 10.1016/j.jpeds.2009.06.044 19840614 PMC3857033

[3] Lucas A Morley R Cole TJ. Adverse neurodevelopmental outcome of moderate neonatal hypoglycaemia. BMJ. (1988) 297:1304–8. 10.1136/bmj.297.6659.1304 2462455 PMC1834933

[4] Duvanel CB Fawer CL Cotting J Hohlfeld P Matthieu JM. Long-term effects of neonatal hypoglycemia on brain growth and psychomotor development in small-for-gestational-age preterm infants. J Pediatr. (1999) 134:492–8. 10.1016/S0022-3476(99)70209-X 10190926

[5] McKinlay CJD Alsweiler JM Anstice NS Burakevych N Chakraborty A Chase JG. Association of neonatal glycemia with neurodevelopmental outcomes at 45 years. JAMA Pediatr. (2017) 171:972–83. 10.1001/jamapediatrics.2017.1579 28783802 PMC5710616

[6] Kinnala A Rikalainen H Lapinleimu H Parkkola R Kormano M Kero P. Cerebral magnetic resonance imaging and ultrasonography findings after neonatal hypoglycemia. Pediatrics. (1999) 103:724–9. 10.1542/peds.103.4.724 10103293

[7] Adamkin DH Polin RA. Imperfect Advice: Neonatal hypoglycemia. J Pediatr. (2016) 176:195–6. 10.1016/j.jpeds.2016.05.051 27297210

[8] Committee on Fetus and Newborn Adamkin DH. Postnatal glucose homeostasis in late-preterm and term infants. Pediatrics. (2011) 127:575–9. 10.1542/peds.2010-3851 21357346

[9] Thornton PS Stanley CA De Leon DD Harris D Haymond MW Hussain K. Recommendations from the Pediatric Endocrine Society for evaluation and management of persistent hypoglycemia in neonates, infants, and children. J Pediatr. (2015) 167:238–45. 10.1016/j.jpeds.2015.03.057 25957977 PMC11891912

[10] Adamkin DH. Neonatal hypoglycemia. Semin Fetal Neonatal Med. (2017) 22:36–41. 10.1016/j.siny.2016.08.007 27605513

[11] Srinivasan G Pildes RS Cattamanchi G Voora S Lilien LD. Plasma glucose values in normal neonates: a new look. J Pediatr. (1986) 109:114–7. 10.1016/S0022-3476(86)80588-1 3723230

[12] Heck LJ Erenberg A. Serum glucose levels in term neonates during the first 48 h of life. J Pediatr. (1987) 110:119–22. 10.1016/S0022-3476(87)80303-7 3794870

[13] Finegold DN Stanley CA Baker L. Glycemic response to glucagon during fasting hypoglycemia: an aid in the diagnosis of hyperinsulinism. J Pediatr. (1980) 96:257–9. 10.1016/S0022-3476(80)80817-1 7351590

[14] Stanley CA Baker L. Hyperinsulinism in infancy: diagnosis by demonstration of abnormal response to fasting hypoglycemia. Pediatrics. (1976) 57:702–11. 10.1542/peds.57.5.702 940710

[15] Stanley CA Anday EK Baker L Delivoria-Papadopolous M. Metabolic fuel and hormone responses to fasting in newborn infants. Pediatrics. (1979) 64:613–9. 10.1542/peds.64.5.613 492835

[16] Brand PL Molenaar NL Kaaijk C Wierenga WS. Neurodevelopmental outcome of hypoglycaemia in healthy, large for gestational age, term newborns. Arch Dis Child. (2005) 90:78–81. 10.1136/adc.2003.039412 15613521 PMC1720084

[17] Hume R McGeechan A Burchell A. Failure to detect preterm infants at risk of hypoglycemia before discharge. J Pediatr. (1999) 134:499–502. 10.1016/S0022-3476(99)70210-6 10190927

[18] Chua KS Tan IK. Plasma glucose measurement with the yellow springs glucose analyzer. Clin Chem. (1978) 24:150–2. 10.1093/clinchem/24.1.150 618647

[19] College of American Pathologists. General Chemistry/Therapeutic Drug Monitoring Survey C-C [Participant Summary]. (2020).

[20] Curme HG Columbus RL Dappen GM Eder TW Fellows WD Figueras J. Multilayer film elements for clinical analysis: general concepts. Clin Chem. (1978) 24:1335–42. 10.1093/clinchem/24.8.1335 679457

[21] Stepman HC Tiikkainen U Stöckl D Vesper HW Edwards SH Laitinen H. Measurements for 8 common analytes in native sera identify inadequate standardization among 6 routine laboratory assays. Clin Chem. (2014) 60:855–63. 10.1373/clinchem.2013.220376 24687951 PMC5699466

[22] Spong CY. Defining “term” pregnancy: recommendations from the Defining “Term” Pregnancy Workgroup. JAMA. (2013) 309:2445–6. 10.1001/jama.2013.6235 23645117

[23] Alexander GR Himes JH Kaufman RB Mor J Kogan M. A United States national reference for fetal growth. Obstet Gynecol. (1996) 87:163–8. 10.1016/0029-7844(95)00386-X 8559516

[24] Ballard JL Khoury JC Wedig K Wang L Eilers-Walsman BL Lipp R. New Ballard score, expanded to include extremely premature infants. J Pediatr. (1991) 119:417–23. 10.1016/S0022-3476(05)82056-6 1880657

[25] Morrison B. Use of the Beckman glucose analyzer for low and high glucose values. Clin Chim Acta. (1972) 42:192. 10.1016/0009-8981(72)90395-6 4654853

[26] Passey RB Gillum RL Fuller JB Urry FM Giles ML. Evaluation and comparison of 10 glucose methods and the reference method recommended in the proposed product class standard (1974). Clin Chem. (1977) 23:131–9. 10.1093/clinchem/23.1.131 832363

[27] Sacks DB Arnold M Bakris GL Bruns DE Horvath AR Kirkman MS. Guidelines and recommendations for laboratory analysis in the diagnosis and management of diabetes mellitus. Clin Chem. (2011) 57:e1–e47. 10.1373/clinchem.2011.163634 21617152

[28] Dickson LM Buchmann EJ Janse Van Rensburg C Norris SA. The impact of differences in plasma glucose between glucose oxidase and hexokinase methods on estimated gestational diabetes mellitus prevalence. Sci Rep. (2019) 9:7238. 10.1038/s41598-019-43665-x 31076622 PMC6510785

[29] Hagvik J. Glucose measurement: time for a gold standard. J Diabetes Sci Technol. (2007) 1:169–72. 10.1177/193229680700100205 19888402 PMC2771464

[30] Pelletier O Arratoon C. Precision of glucose measurements in control sera by isotope dilution/mass spectrometry: proposed definitive method compared with a reference method. Clin Chem. (1987) 33:1397–402. 10.1093/clinchem/33.8.1397 3301068

[31] Altman DG Bland JM. Measurement in Medicine: the analysis of method comparison studies. J R Stat Soc Ser D. (1983) 32:307–17. 10.2307/2987937

[32] Kaiser JR Bai S Rozance PJ. Newborn plasma glucose concentration nadirs by gestational-age group. Neonatology. (2018) 113:353–9. 10.1159/000487222 29510404

[33] Fenton TR Kim JH. A systematic review and meta-analysis to revise the Fenton growth chart for preterm infants. BMC Pediatr. (2013) 13:59. 10.1186/1471-2431-13-59 23601190 PMC3637477

[34] Bonellie S Chalmers J Gray R Greer I Jarvis S Williams C. Centile charts for birthweight for gestational age for Scottish singleton births. BMC Pregnancy Childbirth. (2008) 8:5. 10.1186/1471-2393-8-5 18298810 PMC2268653

[35] Beck RW Riddlesworth T Ruedy K Ahmann A Bergenstal R Haller S. Effect of continuous glucose monitoring on glycemic control in adults with type 1 diabetes using insulin injections: the DIAMOND randomized clinical trial. JAMA. (2017) 317:371–8. 10.1001/jama.2016.19975 28118453

[36] Hume R Burchell A Williams FL Koh DK. Glucose homeostasis in the newborn. Early Hum Dev. (2005) 81:95–101. 10.1016/j.earlhumdev.2004.10.005 15707720

[37] Marconi AM Paolini C Buscaglia M Zerbe G Battaglia FC Pardi G. The impact of gestational age and fetal growth on the maternal-fetal glucose concentration difference. Obstet Gynecol. (1996) 87:937–42. 10.1016/0029-7844(96)00048-8 8649702

